# Effects of air oversaturation and wettability on ultrasonication-generated surface microbubbles and their implications for froth flotation

**DOI:** 10.1016/j.ultsonch.2026.107960

**Published:** 2026-07-14

**Authors:** Ming Xu, Haijun Zhang, Martin Rudolph

**Affiliations:** a Helmholtz-Zentrum Dresden-Rossendorf, Helmholtz Institute Freiberg for Resource Technology, Freiberg 09599, Germany; b School of Chemical Engineering and Technology, China University of Mining & Technology, Xuzhou, Jiangsu 221116, China

**Keywords:** Froth flotation, Surface microbubbles, Ultrasonication, Dissolved-air concentrations, Wettabilities

## Abstract

Surface microbubbles generated during ultrasonic pretreatment can enhance froth flotation. However, the effective utilization of these microbubbles requires air-oversaturated conditions in the liquid after ultrasonication. To verify this requirement, dissolved-air concentrations were measured before and after ultrasonication at different sonication durations, and the evolution of ultrasonication-generated surface microbubbles was monitored on microcavity-containing substrates with different wettabilities. Bubble pick-up and collision experiments were further conducted to evaluate the role of ultrasonication-generated surface microbubbles in particle flotation. Dissolved-air measurements and imaging observations indicate that ultrasonication generates surface microbubbles through two pathways: (i) nucleation of bulk bubbles followed by attachment to solid surfaces, and (ii) in situ nucleation from surface microcavities. The post-ultrasonication evolution of surface microbubbles demonstrates that air oversaturation is the dominant factor controlling the stable surface-microbubble population, whereas microbubbles cannot remain stable under non-oversaturated conditions. By contrast, increasing sonication duration has only a limited effect on the stable bubble population, although it slightly increases the initial number of bubbles. Surface microbubble formation also exhibits strong selectivity toward hydrophobic surfaces. Bubble pick-up and collision experiments demonstrate that ultrasonication-generated surface microbubbles enhance the attachment of hydrophobic particles to carrier bubbles and increase particle loading only under air-oversaturated conditions.

## 1 Introduction

Froth flotation is an economic and efficient method for separating fine mineral particles based on differences in mineral wettability [1]. Generally, bubble-particle interactions during flotation can be separated into three subprocesses: collision, attachment, and detachment. The flotation recovery of fine particles is low due to their low collision and attachment probabilities with coarse carrier bubbles [2]. Surface microbubbles can improve the flotation recovery of fine particles by increasing both the collision and attachment probabilities [3], [4]. In particular, surface microbubbles can enhance particle agglomeration via capillary bridging, increasing the apparent particle size and thereby improving the collision probability with carrier bubbles [5]. Gas nuclei can be trapped in microcavities and crevices on mineral surfaces, serving as nucleation sites [6]. Accordingly, some studies have promoted gas nucleation at these sites by introducing air-oversaturated water and decompressing the flotation system, thereby forming surface microbubbles [7], [8]. Sonication has also been employed as a pretreatment to improve flotation efficiency [9], [10] and has been reported to promote surface microbubble formation on particle surfaces [11], [12].

Ultrasonication can enhance the flotation of mineral particles through multiple mechanisms, including cleaning mineral surfaces [13], increasing surface roughness [14], improving reagent adsorption [15], promoting particle aggregation [16], and generating microbubbles [17]. Ultrafine gangue coatings and oxidation films can be removed by the collapse of cavitation bubbles near mineral surfaces, thereby increasing surface hydrophobicity and enhancing wettability contrast among different mineral species [18], [19]. The high-speed jet induced by cavitation can also increase surface roughness and expose additional active sites for collector adsorption [20]. In addition, acoustic radiation forces can promote bubble-particle aggregation, thereby improving the flotation efficiency of fine particles [5], [21]. By carefully controlling sonication duration and acoustic amplitude, ultrasonication can further enhance particle attachment to bubble surfaces [22]. In practice, applying ultrasonication as a pretreatment is more convenient than applying it during flotation. In situ ultrasonication requires stricter control because it can directly alter bubble-particle interactions [23]. Although some studies have recognized that ultrasound can facilitate surface microbubble formation and thereby enhance flotation [24], the mechanisms by which ultrasound generates surface microbubbles remain insufficiently investigated.

In an acoustic field, rectified diffusion can drive microbubble nucleation and growth [25], [26]. It arises from asymmetric mass transfer across the bubble interface during repeated expansion-compression cycles driven by the pressure wave. During expansion, dissolved gases (and volatile species) diffuse into the bubble, whereas during compression, they diffuse out. However, the net mass flux over one cycle is typically inward because (i) the bubble has a larger interfacial area during expansion, enhancing mass transfer, and (ii) the concentration boundary layer is thinner during expansion and thicker during compression, producing a larger concentration gradient and stronger mass influx at the interface in the expansion phase [27]. As a result, the amount of gas leaving the bubble during compression is smaller than the amount entering during expansion, leading to a cumulative increase in bubble size over successive cycles. Once a bubble reaches a critical size, it may collapse, generating additional nuclei for subsequent nucleation and growth [26]. Acoustic radiation forces can enhance the attachment of ultrasound-generated bulk microbubbles to particle surfaces. Moreover, rectified diffusion can drive bubble nucleation and growth from microcavities and crevices on particle surfaces during ultrasonication.

In mineral flotation, surface microbubbles generated during ultrasonic pretreatment can promote the agglomerate formation and enhance particle attachment to carrier bubbles via capillary bridging [28]. As a result, flotation performance can be enhanced, particularly for fine particles. In general, increasing the size and number of surface microbubbles can strengthen this enhancement [29]. The gas-oversaturation level plays an important role in microbubble generation [30] and is also a key parameter governing the evolution of these microbubbles [31] during flotation. Under undersaturated conditions, microbubbles shrink and dissolve, reducing their size and number, whereas under oversaturated conditions, microbubbles can remain stable and continue to grow. Wettability also plays an important role. Increasing hydrophobicity promotes microbubble attachment to solid surfaces and favors the trapping of gas nuclei in surface microcavities and crevices, which can act as nucleation sites [32]. Consequently, more surface microbubbles tend to form on hydrophobic surfaces. Yang et al. [33] reported that hydrophobic coal and hydrophilic quartz could be separated using only ultrasonication-generated bubbles (without external gas supply): tiny bubbles formed on the coal surfaces, whereas no bubbles formed on hydrophilic quartz in water. However, the effects of surface wettability and gas oversaturation on the formation and stability of ultrasonication-generated surface microbubbles have not been systematically investigated.

In this study, ultrasonic pretreatment was used to generate surface microbubbles on microcavity-containing silicon wafers with different wettability states in air-saturated and air-oversaturated deionized water. Afterwards, their evolution was monitored. The effects of air oversaturation and surface wettability on the post-ultrasonication evolution of surface-microbubble number and size are analyzed, and the underlying mechanisms are discussed. In addition, the influence of ultrasonication-generated surface microbubbles on particle loading on the surface of a coarse carrier bubble is evaluated for particles with different wettability. This study advances understanding of ultrasonication-generated surface microbubble formation and guides their effective use in flotation processes.

## 2 Methods and materials

### 2.1 Materials

Two types of quartz particles were used in this study: hydrophilic and hydrophobic. Pristine pure quartz was purchased from Krantz Mineralienkontor, Germany, and ground to a particle size of 80 µm to 100 μm. Subsequently, these quartz particles were sequentially cleaned with 0.5 mol/L sulfuric acid, 0.5 mol/L sodium hydroxide, and deionized (DI) water [29]. After this cleaning process, the water contact angle measured on a glass slide was approximately 39°, as reported in a previous study [34]. This value was used to represent the contact angle of the cleaned quartz particles. Unless otherwise specified, all contact angles hereafter refer to water contact angles. These particles were therefore treated as hydrophilic quartz particles (HI). After cleaning and drying, the quartz particles were functionalized by esterification with 1-hexanol. The resulting particles are called hydrophobic quartz particles (HO). Detailed descriptions of the cleaning and esterification processes are provided in Sygusch and Rudolph [34]. As reported in that study, the contact angle measured on a glass slide after esterification was ∼85°, and this value was used to represent the contact angle of HO.

Similarly, this study employed two types of silicon wafers: hydrophilic and hydrophobic. Cylindrical microcavities (30 µm in diameter and 30 µm in depth) were fabricated on the wafer surfaces using deep reactive ion etching [31]. The cleaning procedure for the silicon wafers was described in our previous study [29]. The cleaned silicon wafer has a native silicon oxide layer, making its surface chemistry comparable to that of quartz. As reported in that study, the contact angle of the cleaned wafer surface was 39.1 ± 1.7. After cleaning, the wafers were treated with piranha solution, as reported in our previous study [29]. Subsequently, the treated wafers were esterified with 1-hexanol. The contact angle of the esterified wafer surface was 80.6 ± 0.7 [29], which is close to the contact angle used to represent HO.

### 2.2 Water preparation

Two air-saturation states of DI water were prepared in this study. First, Air-saturated water, hereafter referred to as SW, was prepared by equilibrating DI water with atmospheric air at room temperature for 72 h. The room temperature and atmospheric pressure were 20 °C ± 0.5 °C and 9.85 × 10^4^ Pa ± 0.20 × 10^4^ Pa, respectively. Second, Air-oversaturated water, hereafter referred to as OW, was obtained by pressurizing SW with compressed air to a dissolved-air concentration of ∼0.87 mol/m^3^ (corresponding to an oversaturation level of ∼0.12 [31]).

### 2.3 Dissolved-air concentration measurements before and after ultrasonication

For each water type, 100 mL of liquid was placed in a cubic glass cuvette (50 mm × 50 mm × 50 mm). The temperature and dissolved-oxygen concentration were measured before and after ultrasonication for 2 s and 4 s using an optical oxygen sensor (OXYBase, Presen, Germany). In DI water, dissolved oxygen accounts for 35.6 % of the dissolved air on a molar basis, and this fraction is independent of temperature [31]. Therefore, the dissolved-air concentration can be calculated through the measured dissolved-oxygen concentration. Ultrasonic treatment was performed using a horn-type ultrasonic device (SONOPULS HD 2200, BANDELIN, Germany) operated at a frequency of 20 kHz and a nominal power of 60 W. The ultrasonic probe had a diameter of 13  mm, and the probe tip was immersed 6 mm below the air–liquid interface. The difference in water temperature before and after ultrasonication for 2 s and 4 s was less than 1 °C. These ultrasonic parameters were kept constant throughout the study.

### 2.4 Observations for the evolution of surface microbubbles

**Fig.1:**
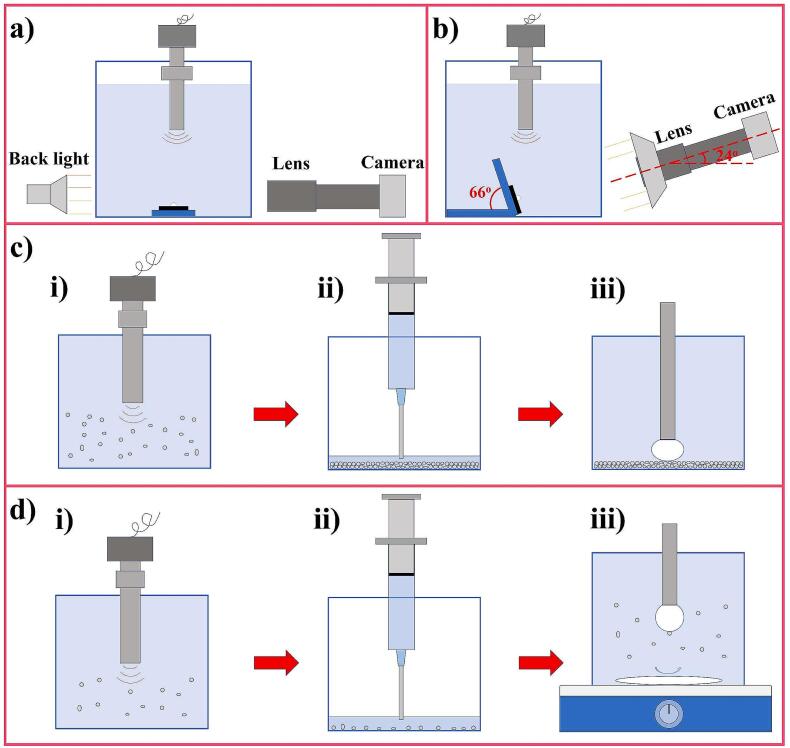
Schematics of the side-view (a) and top-view (b) imaging setups for observing surface microbubbles, and experimental workflows for the bubble pick-up (c) and bubble collision (d) measurements.

In this study, the evolution of surface microbubbles on wafer surfaces with varying wettability during and after ultrasonic treatment for different durations (2 s and 4 s) was monitored. Both top-view and side-view images of the microbubbles were recorded using the imaging system of a contact-angle measurement instrument (OCA50Pro, DataPhysics, Germany). Schematics of the side-view and top-view observation setups are shown in Fig. 1(a) and (b), respectively. In each experiment, 100 mL of the selected type of water was added to the cubic cuvette described in Sec. 2.3.

As shown in Fig. 1(a), the silicon wafer was attached to the bottom of the cuvette. The wafer surface and the central axis of the lens and camera were parallel to the horizontal plane. The light source was placed on the back side of the cuvette, parallel to the horizontal plane. The distance between the ultrasonic probe tip to the wafer center was ∼32 mm.

As shown in Fig. 1(b), the silicon wafer was attached to a sample holder. The wafer surface and the sample holder formed an angle of 66° with the bottom of the cuvette. In addition, the central axis of the lens and camera formed a 24° angle with the horizontal plane. A ring light was mounted around the lens to illuminate the sample surface. In the top-view images, surface microbubbles appear as bright spots because of light reflection by the bubbles. The distance between the ultrasonic probe tip to the wafer center was ∼29 mm.

### 2.5 Bubble pick-up and collision experiments

The OCA50 instrument was additionally used to perform single-bubble pick-up and collision experiments in both SW and OW. These experiments enabled comparison of the attachment behavior of HI and HO to carrier bubbles under different gas-saturation and surface-microbubble conditions, namely SW without ultrasonication-generated surface microbubbles, OW without ultrasonication-generated surface microbubbles, and OW containing ultrasonication-generated surface microbubbles. The ultrasonic treatment duration was fixed at 2 s. The cubic glass cuvette described in Sec. 2.3 was used for both the bubble pick-up and collision experiments.

Fig. 1(c) shows the procedure for the pick-up experiments, which is described as follows: (i) A total of 2 g of either HI or HO was added to a cuvette containing 100 mL of SW or OW, followed by sonication for 2 s. The distance between the ultrasonic probe tip and the particle bed was ∼33 mm. (ii) After 4 min, most particles had settled and the liquid had become clear; a syringe was utilized to remove 90 mL of the upper liquid. This 4 min resting period ensured that, in SW, the ultrasonication-generated surface microbubbles dissolved and disappeared. In contrast, in OW, the generated surface microbubbles remained stable and could continue to grow during this period. (iii) The cuvette was refilled with 90 mL of SW or OW. A glass syringe was used to generate an air bubble with a diameter of (3.5 ± 0.2) mm at the tip of a submerged needle. The air bubble was gradually moved downward to contact the bed of settled particles and then held at the desired position for 10 s to enable particles to attach. To maintain consistent contact conditions, the horizontal position of the needle tip during attachment was precisely marked. After the attachment step, the bubble was slowly moved upward for image acquisition. For each condition, attachment tests were performed at ten different positions on the particle bed.

The process of the collision experiment is shown in Fig. 1(d). The experiment was conducted as follows: (i) A total of 0.3 g of either HI or HO was added to a cuvette containing 100 mL of SW or OW, followed by sonication for 2 s. (ii) After 4 min, a syringe was utilized to remove 90  mL of the upper liquid, and the cuvette was positioned on a magnetic stirrer. (iii) The cuvette was refilled with 90 mL of SW or OW. A glass syringe was used to generate an air bubble with a diameter of (3.5 ± 0.2) mm at the tip of a submerged needle. Following bubble generation, the particles were resuspended using a magnetic stirrer bar operating at 400 rpm for 20 s to facilitate collisions with the bubble. The magnetic stirrer bar had a diameter of 6 mm and a length of 30 mm. The positions of the cuvette and the syringe were precisely marked to maintain the bubble at a fixed location, ensuring reproducible fluid conditions. After stirring had stopped and most particles had settled, images of the bubble were saved for later analysis. For each condition, the collision experiment was conducted seven times.

## 3 Results

### 3.1 Change of dissolved-air concentrations caused by ultrasonication

**Fig. 2:**
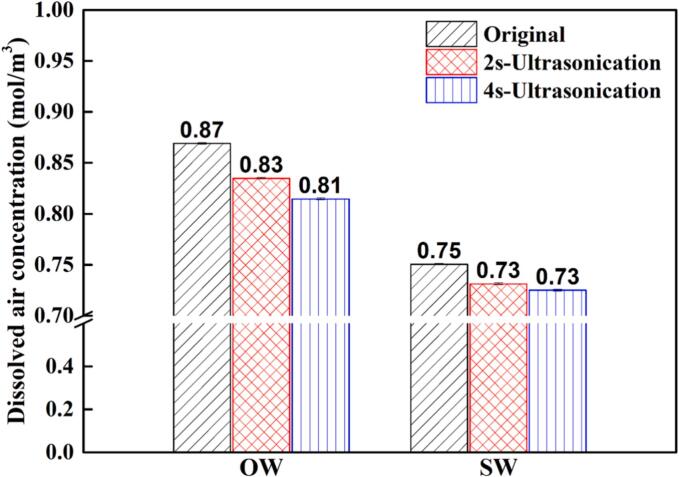
Dissolved-air concentrations in SW and OW measured before and after ultrasonication for 2 s and 4 s. Data are averages of three independent experiments conducted under identical conditions; error bars indicate 95% confidence intervals.

Ultrasonication can induce bulk bubble nucleation, leading to the formation of air nano/microbubbles and a corresponding decrease in dissolved-air concentration [35]. Fig. 2 presents the changes in dissolved-air concentration measured in SW and OW before and after ultrasonic treatment with durations of 2 s and 4 s. After sonication, the temperature difference between the 2 s and 4 s treatments was negligible; therefore, a water temperature of 21 °C was assumed for all cases after ultrasonication. At this temperature and an atmospheric pressure of 9.85 × 10^4^ Pa, the theoretical air saturation concentration in water is 0.76 mol/m^3^ [36]. Before ultrasonication, the dissolved-air concentration in SW calculated from the measured dissolved-oxygen concentration was 0.75 mol/m^3^, which is slightly lower than the theoretical value of 0.78 mol/m^3^ at 20 °C and 9.85 × 10^4^ Pa [36]. This small deviation may be attributed to slight fluctuations in room temperature and atmospheric pressure during the equilibration period. Nevertheless, the measured dissolved-air concentration was close to the theoretical saturation value; therefore, the water was still considered air-saturated. As illustrated, ultrasonication reduces the dissolved-air concentration, with the rate of reduction gradually slowing as ultrasonication time increases. Furthermore, the decrease in dissolved-air concentration is more pronounced in OW than in SW. After ultrasonication, SW becomes undersaturated, whereas OW remains oversaturated.

During ultrasonication, the dissolved-air concentration decreases because a fraction of the dissolved-air molecules transfers to the gas phase by entering freshly nucleated microbubbles. However, while microbubbles remain suspended in the liquid before reaching the upper air–liquid interface, gas can also dissolve back into the liquid due to the high Laplace pressure at the microbubble interface. The dissolution rate is governed by the gradient of dissolved-air concentration between the bubble surface and the bulk liquid. Consequently, a lower bulk dissolved-air concentration produces a larger gradient and a higher dissolution flux. Hence, a higher fraction of gas in the microbubbles returns to the liquid when the initial dissolved-air concentration is lower. This explains why the reduction rate decreases as ultrasonication time increases, and why the net reduction in dissolved-air is larger in OW than in SW. The dissolved-air concentration after ultrasonication ultimately determines whether surface microbubbles dissolve or continue to grow.

### 3.2 Impacts of oversaturation on the formation and evolution of surface microbubbles

**Fig. 3:**
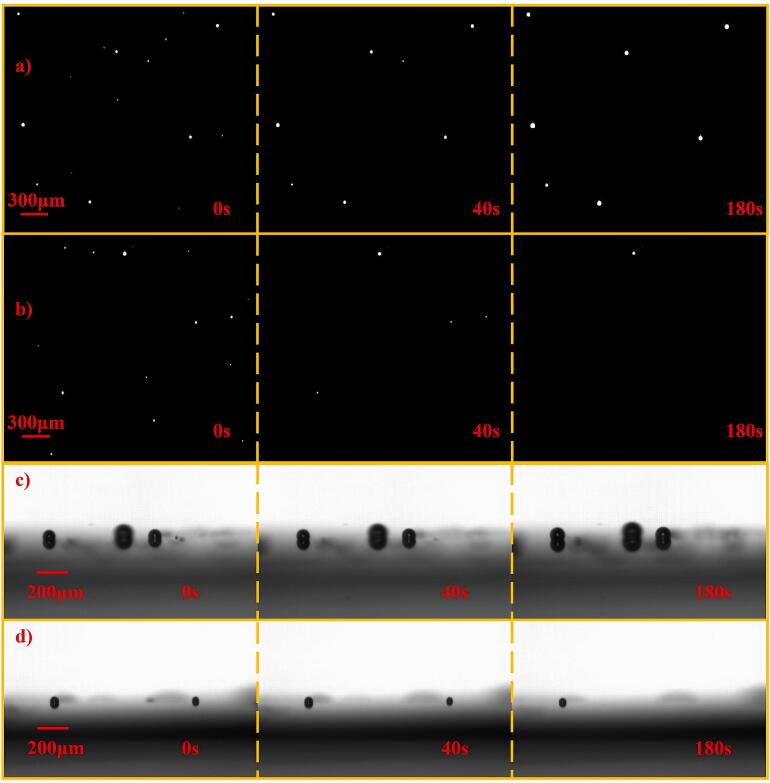
Top-view images of surface microbubbles on a hydrophobic surface in OW (a) and SW (b), and side-view images in OW (c) and SW (d), acquired at 0 s, 40 s, and 180 s after ultrasonication.

For effective application, surface microbubbles must remain stable during post-ultrasonication processes, and their stability is primarily determined by dissolved-air concentration. Fig. 3(a) and (b) show top-view snapshots of hydrophobic surfaces at 0 s, 40 s, and 180 s after ultrasonic pretreatment in OW and SW, respectively. The full top-view field of view is 4.7 mm × 2.5 mm. To better illustrate the evolution of surface microbubbles, cropped regions of 2.8 mm × 2.5 mm are enlarged and displayed in Fig. 3(a) and (b). Fig. 3(c) and (d) present the side-view snapshots under the same conditions.

As shown in Fig. 3(a), in OW, some small bright spots–representing fine bubbles–gradually shrink and disappear, while larger bright spots–corresponding to bigger bubbles–tend to grow over time. In contrast, Fig. 3(b) shows that in SW, all bright spots gradually shrink, with only a few remaining after 180 s. The side-view snapshots display the same trend. Moreover, the observed bubble contact angles are comparable to those measured using water droplets. No surface microbubbles are observed before ultrasonication; thus, all surface microbubbles are generated during ultrasonication treatment.

The dissolved-air concentration at the bubble surface, *c_s_*, can be calculated from Henry’s law, as:

(1) $$c_{s}={K^{H,cp}P_{b}=K^{H,cp}\left(P_{\mathrm{at}}+\frac{2\sigma }{r}\right)=c}_{\mathrm{sat},b}\left(1+\frac{2\sigma }{rP_{\mathrm{at}}}\right)$$

where *K^H,cp^* is Henry’s constant, *P_b_* is the pressure inside the bubble, *σ* is the air–liquid surface tension, *r* is the bubble radius, and *P_at_* is the atmospheric pressure. Here, c*_sat,b_* = *K^H,cp^* × *P_at_* is the air saturation concentration in the bulk water. For a bubble to remain stable (i.e., not dissolve), the bulk dissolved-air concentration *c*_∞_, must satisfy *c*_∞_ ≥ *c_s_*. Therefore, the threshold radius *r_s_* for stability (and potential growth) can be obtained from *c*_∞_ and *c_sat,b_*, as:

(2) $$r_{s}=\frac{2\sigma }{P_{\mathrm{at}}\left(\frac{c_{\infty }}{c_{\mathrm{sat},b}}-1\right)}$$

Using Eq. (2), the threshold radius in OW after 2 s of ultrasonication is estimated to be *r_s_* ≈ 16 μm, based on *σ* = 72 mN/m, *P_at_* = 9.85 × 10^4^ pa, *c_∞_* = 0.83 mol/m^3^ (Fig. 2), and *c_sat,b_* = 0.76 mol/m^3^. In contrast, for SW after 2 s of ultrasonication, the dissolved-air concentration is insufficient to stabilize surface microbubbles, and they therefore tend to dissolve.

**Fig. 4:**
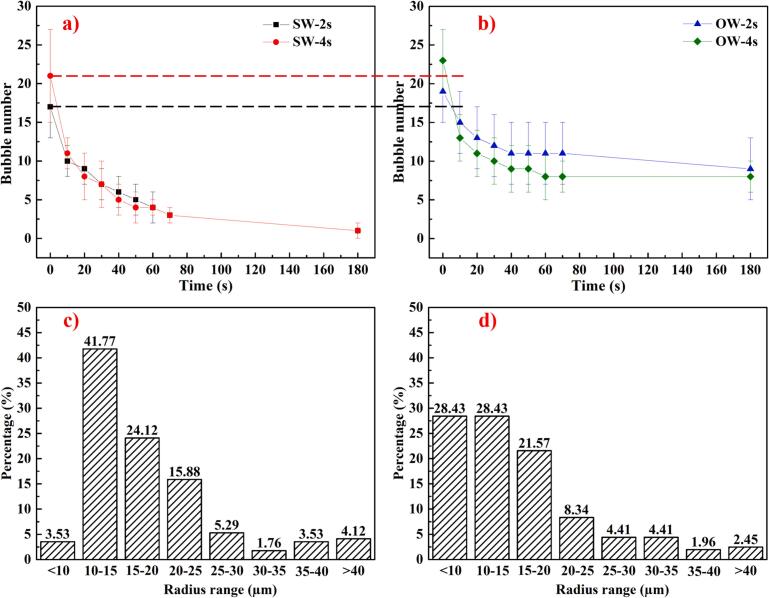
Time evolution of the surface bubble number on a hydrophobic surface after 2 s and 4 s ultrasonication in (a) SW and (b) OW. Data show averages of nine independent experiments conducted under identical conditions; error bars indicate 95% confidence intervals. Radius distributions of surface bubbles formed on hydrophobic surfaces in OW at 0 s after ultrasonication for 2 s (c) and 4 s (d); the distributions were calculated using all bubbles detected in nine independent experiments under each condition.

Fig. 4 shows the variations in bubble numbers on hydrophobic surfaces in SW (a) and OW (b) over time after ultrasonic pretreatment of different durations (2 s and 4 s), based on top-view snapshots. The reported bubble numbers are averages over nine experiments conducted under identical conditions. To capture more bubbles for statistical analysis, the top-view field of view was expanded to 4.7 mm × 2.5 mm. As shown in the figure, the initial average bubble numbers in OW (19 for 2 s ultrasonication and 23 for 4 s) are slightly higher than those in SW (17 for 2 s and 21 for 4 s). However, the final average bubble numbers at 180 s in OW (9 for 2 s and 8 for 4 s) are significantly higher than those in SW, where only one bubble remains for both 2 s and 4 s ultrasonication. Moreover, the initial average bubble number is consistently higher with 4 s of ultrasonication than with 2 s.

Fig. 4(c) and (d) show the radius distributions of surface microbubbles formed on hydrophobic surfaces in OW at 0 s after ultrasonication for 2 s and 4 s, respectively. For each condition, the percentage was calculated by dividing the number of bubbles within each radius range by the total number of bubbles detected across nine independent experiments. The method used to calculate the bubble radius from the size of the bright spots is described in the Supporting Information. The results indicate that extending the ultrasonication duration increased the proportion of bubbles with radii smaller than 10 μm.

The number of nucleated microbubbles is mainly controlled by the transiently high local air oversaturation induced by the substantial pressure drop near the ultrasonic probe, which explains the small difference in the initial bubble number between OW and SW. Extending ultrasonication time increases the number of nucleation events and, consequently, the initial bubble number. However, longer ultrasonication also results in a lower dissolved-air concentration, thereby increasing the stability threshold radius *r_s_*. For example, Eq. (2) predicts that *r_s_* in OW increases from ∼16 μm after 2 s of ultrasonication to ∼22 μm after 4 s, which disfavors the persistence of smaller bubbles and reduces the number of stable bubbles.

For hydrophobic surfaces in OW at 0 s after 2 s of ultrasonication, the maximum radius of bubbles that subsequently dissolved and the minimum radius of bubbles that subsequently grew were 15.4 ± 2.4 μm and 16.3 ± 2.4 μm, respectively, averaged over nine independent experiments conducted under identical conditions. Therefore, *r_s_* should lie between these two radii. After 4 s of ultrasonication, the corresponding maximum and minimum radii increased to 16.6 ± 3.9 μm and 18.0 ± 3.2 μm, respectively. The deviation from the theoretical value, especially after 4 s of ultrasonication, may be partly attributed to the use of the bulk-averaged dissolved-air concentration (Fig. 2) in calculating *r_s_*. In contrast, the local dissolved-air concentration near surface microbubbles may have been higher than the bulk value due to the dissolution of nearby fine microbubbles. This could lead to an overestimation of the theoretical threshold radius. Uncertainties associated with bubble-size estimation may also contribute to this deviation. Nevertheless, these results still indicate that relatively larger bubbles can dissolve after longer ultrasonication. Overall, these results indicate that air oversaturation is essential for retaining surface microbubbles generated by ultrasonication.

### 3.3 Selective formation of surface microbubbles by ultrasonication

**Fig. 5:**
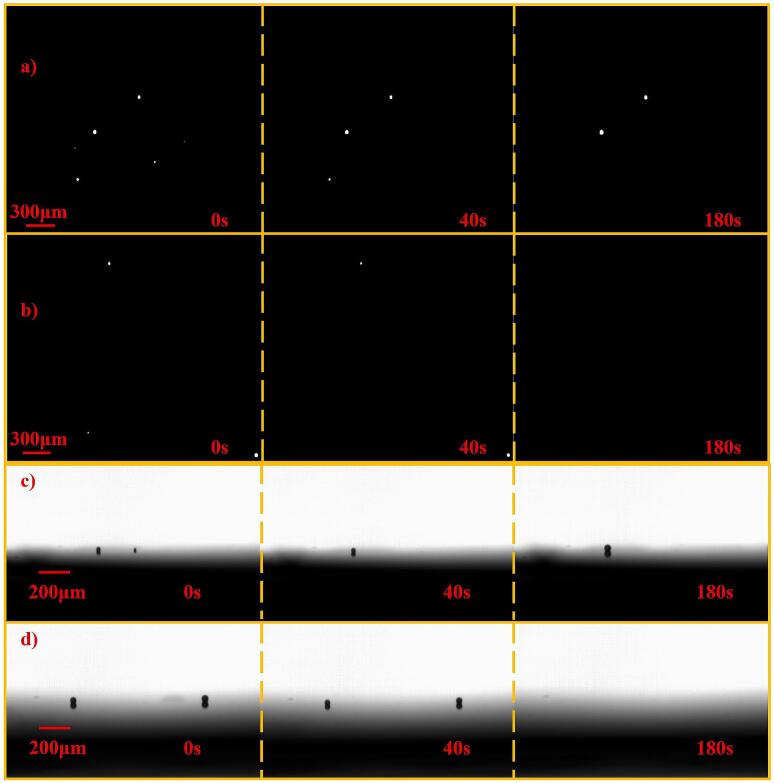
Top-view images of surface microbubbles on a hydrophilic surface in OW (a) and SW (b), and side-view images in OW (c) and SW (d), acquired at 0 s, 40 s, and 180 s after ultrasonication.

In addition to oversaturation, surface wettability significantly affects the initial number of surface microbubbles, as it governs the attachment of bulk-nucleated microbubbles to solid surfaces under acoustic radiation force. Fig. 5(a) and (b) display top-view snapshots of hydrophilic surfaces taken at 0 s, 40 s, and 180 s after ultrasonic pretreatment in OW and SW, respectively. The top-view field of view is the same as in Fig. 4(a) and (b). Fig. 5(c) and (d) show the side-view snapshots under the same conditions. Similar to the results on hydrophobic surfaces, some larger bubbles continue to grow in OW, while smaller microbubbles shrink and disappear. In SW, all bubbles gradually shrink and vanish. On hydrophilic surfaces, the bubble contact angles also match those measured with water droplets.

**Fig. 6:**
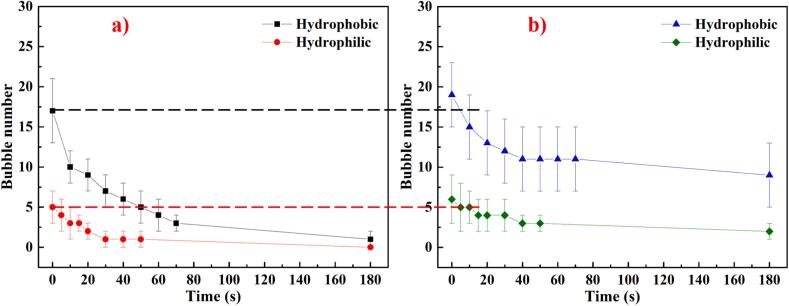
Time evolution of the surface bubble number on hydrophobic and hydrophilic surfaces after 2 s ultrasonication in (a) SW and (b) OW. Data show averages of nine independent experiments conducted under identical conditions; error bars indicate 95% confidence intervals.

Fig. 6 compares the evolution of bubble numbers on hydrophilic and hydrophobic surfaces in SW (a) and OW (b) over time after 2 s of ultrasonic pretreatment, based on top-view snapshots. The reported number is averaged over nine experiments under identical conditions. To capture more bubbles for statistical analysis, the top-view field of view was expanded to 4.7 mm × 2.5 mm. From the comparison, it is evident that the initial average bubble number on the hydrophobic surface (19 in OW and 17 in SW) is significantly higher than that on the hydrophilic surface (6 in OW and 5 in SW). Furthermore, the number of stable bubbles on the hydrophobic surface at 180 s (9 in OW and 1 in SW) also exceeds that on the hydrophilic surface (2 in OW and none in SW). These results indicate that surface microbubbles exhibit preferential attachment to hydrophobic surfaces, likely due to a lower adhesion energy barrier.

**Fig. 7:**
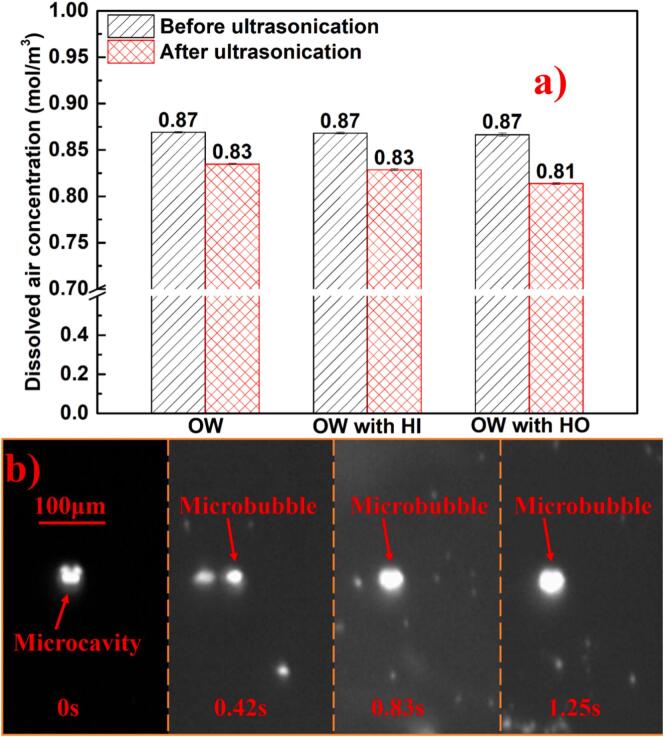
(a) Dissolved-air concentrations in OW measured before and after 2 s ultrasonication for three conditions: particle-free water and suspensions containing HI or HO. Data are based on three independent experiments under identical conditions; error bars indicate 95% confidence intervals. (b) Representative image sequence showing microbubble nucleation and growth at a microcavity on a hydrophobic wafer surface during ultrasonication.

To further assess the selectivity of ultrasonication-generated surface microbubbles, ultrasonication tests were conducted in 100 mL of OW containing 2 g of HO or HI. Fig. 7(a) shows the dissolved-air concentration measured before and after 2 s of ultrasonication for three cases: particle-free water (i.e., pure water) and suspensions containing HO or HI. As shown, the reduction in dissolved-air concentration is markedly larger in the presence of HO than in the presence of HI, while the HI case is comparable to that of pure water. This behavior likely arises because microbubbles preferentially attach to HO and continue to grow, enhancing gas transfer from the liquid to the bubble phase and thereby producing a larger decrease in dissolved air.

In addition, bubble nucleation and growth from surface microcavities may contribute to this difference. Compared with HI, HO likely provides more nucleation sites during ultrasonication because more air is trapped in natural microcavities and crevices. Consequently, more surface microbubbles form on HO [37]. Fig. 7(b) shows a representative image sequence of an etched microcavity on a hydrophobic silicon wafer in SW during ultrasonication, illustrating microbubble nucleation and subsequent growth at the cavity.

### 3.4 Bubble pick-up and collision experiments

Considering the influence of air oversaturation and surface wettability on microbubble formation, the combination of ultrasonication and OW is proposed as a strategy to improve flotation efficiency. Bubble collision and pick-up experiments were conducted to investigate the effects of surface microbubbles generated by ultrasonication on collision and attachment processes in flotation. To evaluate these effects, the coverage percentage of particles on the bubble (*P*) was analyzed using *ImageJ* software and defined as the ratio of the particle-covered area to the overall bubble surface area [29], [38]. Details of *P* determination are provided in our previous study [29].

**Fig. 8:**
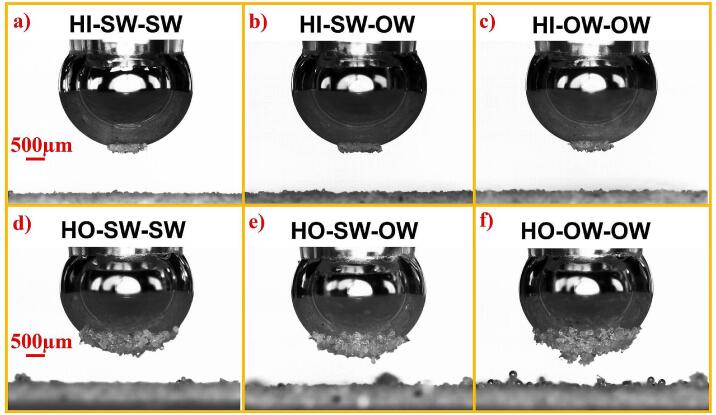
Representative images from the bubble pick-up experiments for HI and HO in SW and OW: (a) HI-SW-SW; (b) HI-SW-OW; (c) HI-OW-OW; (d) HO-SW-SW; (e) HO-SW-OW; (f) HO-OW-OW. The first and second “SW/OW” labels indicate the SW/OW used in steps (i) and (iii) of the procedure in Fig. 1(c), respectively.

Fig. 8(a)–(f) show the representative images of bubble pick-up experiments for HI and HO in SW and OW. As shown in the figures, the first and second “SW/OW” labels denote the SW/OW used in steps (i) and (iii) of the procedure in Fig. 1(c), respectively. Accordingly, “SW-SW”, “SW-OW”, and “OW-OW” represent SW without ultrasonication-generated surface microbubbles, OW without ultrasonication-generated surface microbubbles, and OW with ultrasonication-generated surface microbubbles, respectively. As shown, the particle load of HI exhibits no appreciable differences among the tested conditions. In contrast, for HO, a significant increase in particle loading is observed only when OW is used in both steps.

**Fig. 9:**
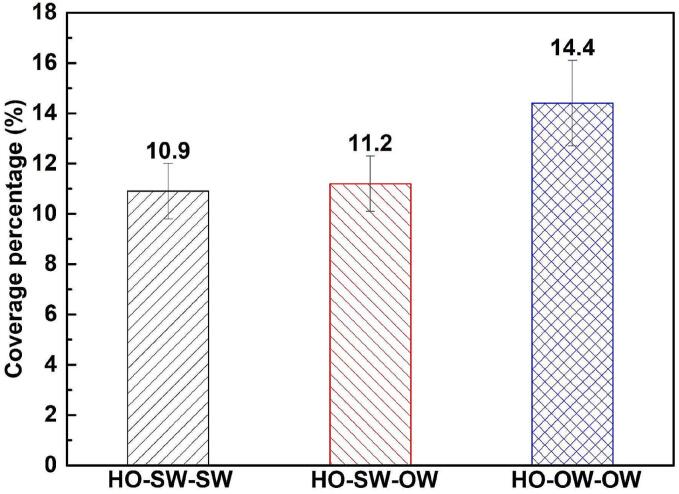
Average coverage percentage of particles on the carrier bubble (*P*) for HO in the bubble pick-up experiments conducted in SW and OW. The first and second “SW/OW” labels indicate the SW/OW used in steps (i) and (iii) of the procedure in Fig. 1(c), respectively. Data are based on ten independent experiments under identical conditions; error bars indicate 95% confidence intervals.

The corresponding *P* values for HO are summarized in Fig. 9. Specifically, *P* for HO with OW used in both steps reaches 14.4 %, which is significantly higher than in the other two cases, both of which are similar ∼11%. For HO, using OW only in step (iii) is insufficient to generate a substantial population of surface microbubbles and therefore does not enhance attachment to the carrier bubble. Accordingly, a pronounced enhancement is observed only when OW is used in both steps, where ultrasonication promotes surface-microbubble formation and air oversaturation maintains their stability. For HI, although a few microbubbles can remain stable when OW is used in both steps, their number is too small to enhance attachment measurably.

**Fig. 10:**
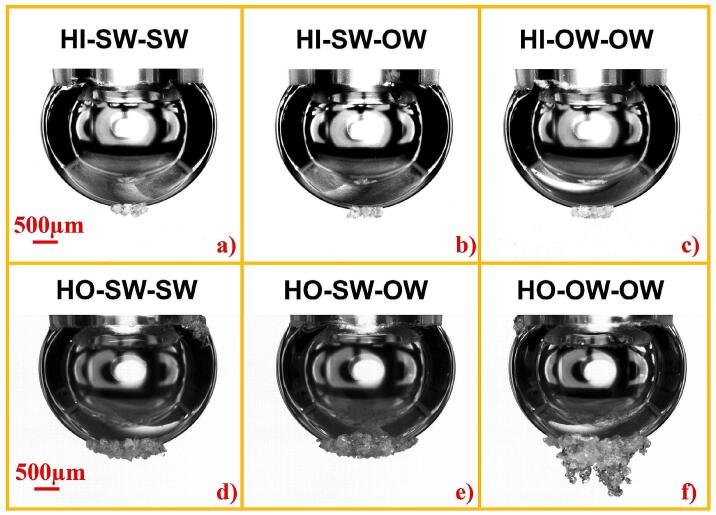
Typical images from the bubble collision experiments for HI and HO in SW and OW: (a) HI-SW-SW; (b) HI-SW-OW; (c) HI-OW-OW; (d) HO-SW-SW; (e) HO-SW-OW; (f) HO-OW-OW. The first and second “SW/OW” labels indicate the SW/OW used in steps (i) and (iii) of the procedure in Fig. 1(d), respectively.

Fig. 10(a)–(f) show typical images from the bubble collision experiments with HI and HO. As shown in the figures, the first and second “SW/OW” labels denote the SW/OW used in steps (i) and (iii) of the procedure in Fig. 1(d), respectively. For HI, only limited attachment to the carrier bubble surface was observed. Moreover, the particle load shows no significant differences among the tested conditions. In contrast, for HO, the particle load increases markedly only when OW was used in both steps, accompanied by the formation of microbubble-particle agglomerates; such agglomeration is not observed with the other two conditions, which exhibit similar particle loads. Because the formation of particle-microbubble agglomerates made it difficult to quantify particle loading using the P value, P values were not compared in the bubble collision experiments. Ultrasonication-generated surface microbubbles promote microbubble-particle agglomeration during agitation, effectively increasing the apparent particle size. In this process, particles bearing surface microbubbles can adhere to other particles via capillary bridging mediated by surface microbubbles [39]. This mechanism is expected to enhance flotation recovery, particularly for fine particles. Overall, the combination of ultrasonication and air oversaturation offers a clear advantage for improving flotation recovery and selectivity for fine hydrophobic particles.

## 4 Conclusions

The effects of air oversaturation and surface wettability on ultrasonication-induced flotation enhancement have not yet been fully clarified. This study elucidates how these two factors regulate the formation and stability of ultrasonication-generated surface microbubbles, based on dissolved-air concentration measurements before and after ultrasonication and observations of the post-sonication evolution of surface-bubble populations. The impacts of ultrasonication-generated surface microbubbles on flotation were further examined using bubble pick-up and collision experiments. The main conclusions are as follows:

(1) Under non-oversaturated conditions, surface microbubbles generated by ultrasonication dissolve rapidly because of their high Laplace pressure. Air oversaturation suppresses bubble dissolution and therefore governs the number of microbubbles that remain stable after ultrasonication. Extending the ultrasonication duration only slightly increases the initial bubble number and may even marginally reduce the final stable bubble population because of stronger dissolved-air depletion.
(2) Ultrasonication-generated surface microbubbles exhibit strong selectivity toward hydrophobic substrates and particles. This selectivity is likely attributable to: (i) the lower energy barrier for bubble adhesion on hydrophobic surfaces, and (ii) the greater tendency of hydrophobic surfaces to retain air in microcavities and crevices, which provides additional gas nucleation sites for surface microbubble formation.
(3) Bubble pick-up and collision experiments demonstrate that the enhancement of hydrophobic particle loading occurs only when ultrasonication is combined with air oversaturation, because many ultrasonication-generated surface microbubbles remain stable under these conditions. These surface microbubbles facilitate the formation of particle agglomerates, thereby increasing particle loading during bubble collision experiments. No measurable improvement is observed for hydrophilic particles under the same conditions.

Overall, the combination of ultrasonication and air oversaturation is expected to improve flotation recovery and selectivity of fine hydrophobic particles. Future studies and applications should explicitly consider, control, and report the air-oversaturation levels in flotation pulps when evaluating ultrasonication-induced enhancement of froth flotation.

## CRediT authorship contribution statement

**Ming Xu:** Writing – review & editing, Writing – original draft, Methodology, Investigation, Formal analysis, Data curation, Conceptualization. **Haijun Zhang:** Writing – review & editing, Supervision. **Martin Rudolph:** Writing – review & editing, Supervision, Funding acquisition, Conceptualization.

## Declaration of competing interest

The authors declare that they have no known competing financial interests or personal relationships that could have appeared to influence the work reported in this paper.

## Appendix A Supplementary material

Supplementary Data 1 Bubble size determination.
