# Supplementary material for: Effects of air oversaturation and wettability on ultrasonication-generated surface microbubbles and their implications for froth flotation
Source: Ultrason Sonochem. 2026 Jul 14;132:107960. doi: 10.1016/j.ultsonch.2026.107960 (PMC13393165; doi:10.1016/j.ultsonch.2026.107960)
Supplement: Supplementary Data 1 — Bubble size determination. [file mmc1.docx]

**Effects of Air Oversaturation and Wettability on Ultrasonication-Generated Surface Microbubbles and Their Implications for Froth Flotation**

**Determination of surface microbubble size from top-view images.**


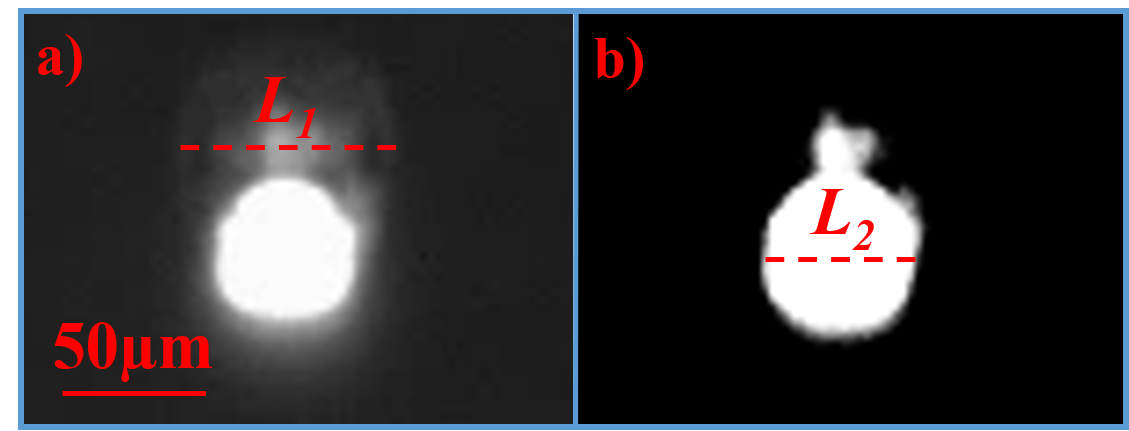


Fig. S1. (a) Original image of a relatively large surface microbubble, where *L_1_* denotes the bubble diameter measured from the bubble contour. (b) Corresponding contrast-enhanced image, where *L_2_* denotes the width of the bright spot used for bubble-size estimation.

In the original top-view images, the contours of some relatively large surface microbubbles could be distinguished, as shown in Fig. S1(a), allowing their diameters, denoted as *L_1_*, to be measured directly. However, for most bubbles, especially smaller ones, the bubble contours were not sufficiently clear for direct diameter measurement, whereas their bright spots could be more readily identified after contrast enhancement. Therefore, the corresponding contrast-enhanced images were used to determine the width of the bright spot, denoted as *L_2_*, using MATLAB, as shown in Fig. S1(b). Based on three individual surface microbubbles for which both *L_1_* and *L_2_* could be measured, the average *L_1_*/*L_2_* ratio was 1.39 ± 0.31. Accordingly, the bubble diameters were estimated by multiplying the bright-spot widths measured from the contrast-enhanced images by 1.39.
